# Perspectives on complement and phagocytic cell responses to nanoparticles: From fundamentals to adverse reactions^☆^

**DOI:** 10.1016/j.jconrel.2023.02.022

**Published:** 2023-03-02

**Authors:** S. Moein Moghimi, Hajira B. Haroon, Anan Yaghmur, A. Christy Hunter, Emanuele Papini, Z. Shadi Farhangrazi, Dmitri Simberg, Panagiotis N. Trohopoulos

**Affiliations:** a School of Pharmacy, Newcastle University, Newcastle upon Tyne NE1 7RU, UK; b Translational and Clinical Research Institute, Faculty of Health and Medical Sciences, Newcastle University, Newcastle upon Tyne NE2 4HH, UK; c Colorado Center for Nanomedicine and Nanosafety, University of Colorado Anschutz Medical Center, Aurora, CO, USA; d Department of Pharmacy, Faculty of Health and Medical Sciences, University of Copenhagen, 2100 Copenhagen Ø, Denmark; e School of Pharmacy, College of Science, University of Lincoln, Lincoln LN6 7TS, UK; f Department of Biomedical Sciences, University of Padua, Padua 35121, Italy; g S. M. Discovery Group Inc., Centennial, CO, USA; h S. M. Discovery Group Ltd., Durham, UK; i Translational Bio-Nanosciences Laboratory, Department of Pharmaceutical Sciences, Skaggs School of Pharmacy, University of Colorado Anschutz Medical Center, Aurora, CO, USA; j CosmoPHOS Ltd, 77 Tsimiski Street, GR-54622 Thessaloniki, Greece

**Keywords:** Adverse reactions, Complement system, Dendrimers, Macrophage, Nanoparticles, NanoLigand carriers, Opsonisation, Pseudoviral nanoparticles, phagocytosis

## Abstract

The complement system, professional phagocytes and other cells such as Natural killer cells and mast cells are among the important components of the innate arm of the immune system. These constituents provide an orchestrated array of defences and responses against tissue injury and foreign particles, including nanopharmaceuticals. While interception of nanopharmaceuticals by the immune system is beneficial for immuno-modulation and treatment of phagocytic cell disorders, it is imperative to understand the multifaceted mechanisms by which nanopharmaceuticals interacts with the immune system and evaluate the subsequent balance of beneficial versus adverse reactions. An example of the latter is adverse infusion reactions to regulatory-approved nanopharmaceuticals seen in human subjects. Here, we discuss collective opinions and findings from our laboratories in mapping nanoparticle-mediated complement and leucocyte/macrophage responses.

## 1. Introduction

Innate (or nonspecific) immunity encompasses anatomical barriers (e.g., mechanical barrier of the skin, the mucous-membrane barrier, the endothelial barrier), physiological parameters (e.g., temperature, acidic pH), physiological clearance processes (e.g., mucocilliary escalator, phagocytic/endocytic barrier offered by blood monocytes, poly-morphonuclear leucocytes, tissue macrophages, dendritic cells, and microfold cells in the gut-associated lymphoid tissue of the Peyer’s patches in the small intestine), protective enzymes (e.g., lysozyme), enzymatic cascades (e.g., the complement system, the contact system), and the inflammatory barrier (e.g., cytokine- and other mediator-driven responses) [1,2]. Innate immunity provides a rapid and orchestrated array of defences against foreign intruders (e.g., pathogens, particles of non-biological origin) and tissue injury. For instance, macrophages are widely distributed throughout the body and strategically placed in many tissues to not only intercept foreign particles and cell debris through phagocytic uptake and lysosomal destruction, but they also contribute to homeostasis through interaction and communication with a wide range of cells and extracellular elements [3]. Thus, it is not surprising to see that macrophages orchestrate many diseases, while dysfunctional macrophages cause severe conditions [4–6]. The propensity of macrophages for phagocytosis arises from their extensive receptor repertoire (e.g., scavenger, Fc, complement and sugar receptors) [3]. This offers diversity in materials recognition, efficient clearance (including the ability to sense spatiotemporal changes on particle surfaces in biological milieu) and signalling responses. Furthermore, the complement system, which is a large family of soluble and membrane-bound proteins, on activation renders most pathogens and organic/inorganic particles susceptible to phagocytosis through opsonisation by the third complement protein (C3) [7–9]. In addition to C3-derived opsonins (e.g., C3b and iC3b), complement pattern-recognition molecules C1q, mannose-binding lectin (MBL) and ficolins may also opsonise nanoparticles, pathogens and apoptotic cells and promote their recognition by effector cells [10–12]. Activation of the terminal pathway of the complement system results in the formation of the C5b-9 lytic complex and its assembly on cell membranes results in transmembrane channels and induces cell death [7]. On the other hand, membrane assembly of sublytic C5b-9 modulate inflammation by promoting cell proliferation and by rescuing apoptotic cells [13]. There is also an intricate relationship between the complement system and the contact system (coagulation) as well as the adaptive arm of the immune system [7,14]. Unlike macrophages, neutrophils further employ extracellular traps, which consist of a network of chromatin fibres decorated with anti-microbial peptides, for killing pathogens that are often too large to be phagocytosed [15]. On the other hand, Natural killer (NK) cells, a group of innate lym-phocytes, are cytotoxic cells and through the release of perforins and granzymes contribute to the destruction of cells infected by viruses as well as in the rejection of tumour cells [16]. NK cells, also, depending on their type, selectively produce interferon-γ and a wide range of cytokines such as interleukin (IL)-4 and −17 that direct a local immune regulation. Mast cells, a type of granulocyte derived from the myeloid stem cells, are long-lived tissue-resident cells that play important roles in many inflammatory settings including infection and injury [17].

Engineered nanoparticles hold promise for wide range of therapeutic, diagnostic and theranostic applications [18,19]. Thus, interception of nanoparticles by different elements of the immune system could bring many beneficial effects [20–22]. For instance, the propensity of the mononuclear phagocytes to ingest therapeutic nanoparticles is advantageous not only for combating persistent infections and treating genetic disorders in these cells, but also for macrophage “reprogramming” within the context of pro- and anti-inflammatory responses, since macrophage polarisation is plastic and reversible [20,23,24]. However, nanoparticle targeting of macrophage subpopulations over the course of infections, chronic inflammatory diseases, neurodegenerative diseases, cancer, tissue injury and repair still require a better understanding of macrophage cell biology and a more precise molecular endotyping. On the other hand, through multifaceted processes and mechanisms, nanoparticle interactions with specific arms of the immune system could also trigger adverse reactions [21,25]. One important example is infusion reactions to regulatory-approved nanopharmaceuticals in human subjects [26]. Thus, it is imperative to understand intricate and multifaceted mechanisms, by which nanoparticles, depending on their phys-icochemical characteristics, interact with soluble and cellular elements of the innate immune system, generate synergy and trigger adverse reactions. Here, we explicitly focus and provide a brief overview of holistic efforts from our laboratories in the nanoparticle-mediated complement activation and opsonisation mechanisms as well as the global leucocyte/macrophage responses to nanoparticles.

## 2. Complement responses to nanomaterials, nanopharmaceuticals and polymers

Early studies with liposomes and polymeric nanoparticles outlined the importance of particle curvature and surface properties in complement activation; however, the underlying mechanisms were poorly understood [27–33]. Later, demonstration of complement activation by Doxil^®^ (a long-circulating liposome formulation with encapsulated doxorubicin) [34] generated much interest in studying complement activation properties of different nano- and micro-particles (reviewed in [9,22,35,36]). Since then, this trend has continued with a diverse range of organic and inorganic materials [37–43]. Our own efforts in complement mapping have included lipid nanoparticles (including liposomes of different phospholipid composition and bilayer characteristics, and a wide range of non-lamellar liquid crystalline aqueous nanodispersions such as cubosomes and hexosomes), tumour cell-derived exosomes, polymeric nanoparticles, micelles, metallic (e.g., iron oxide) nanoparticles, dendrimers, hydrogels, carbon nanotubes, graphene oxide, and archaeal viruses (reviewed in [9,22,36]). The mechanisms by which synthetic particles trigger complement activation are complex and multi-parametric, and we recently reviewed these [36]. However, among the important findings was realisation of the role of particle curvature in modulating antibody clustering (as in IgG) and straining (as in IgM) on surfaces in relation to antibody-orchestrated complement responses [44,45]. Another exciting finding was modulation of complement responses by surface projected polymer chains on polymeric nanospheres [46]. For instance, adsorption of selected block copolymers of poloxamine and poloxamer series on to the surface of polystyrene nanospheres reduces protein adsorption and diminishes complement activation in human serum when compared to uncoated nanoparticles. Intriguingly, changes in copolymer conformation from “mushroom” to “brush” shift the mode of complement activation from classical to lectin pathway [46]. The role of polymer conformation in complement pathway switching was subsequently tested and observed with different nanosystems and polymer types [47,48].

Incorporation of methoxypoly(ethylene glycol) (mPEG)-phospholipids into the liposomal bilayer also confers protection to rapid vesicle clearance by mononuclear phagocytic cells, partly by suppressing protein adsorption and blood opsonisation events (reviewed in [49]). This approach led to the development of Doxil^®^, but complement activation by Doxil^®^ was an unexpected finding [34,50]. To elaborate on complement activation mechanisms by Doxil^®^, our studies identified a role for the anionic phosphate oxygen of the mPEG-lipid moiety in initiating complement activation by PEGylated liposomes [51]. Through methylation of the phosphate oxygen we overcome complement activation [51]. Later, in sterically stabilising a range of non-lamellar liquid crystalline aqueous nanodispersions with non-ionic PEG-lipids, we realised that bilayer properties also modulate complement responses, presumably by affecting PEG mobility and stretching [52]. Considering that PEG is a widely used material in many pharmaceutical preparations, we further questioned whether near-monodisperse endotoxin-free PEG solutions could also trigger complement activation. Our studies revealed complement activation on a time-scale of minutes, but in a PEG concentration- and molecular mass-dependent manner [53]. Complement activation was either exclusively through the alternative pathway or through both lectin and alternative pathways [53]. Realisation of complement activation by PEGs was taken advantage of to generate an accelerated mouse model of choroidal neovascularisation (CNV) and study age-related macular degeneration [54]. Indeed, sub-retinal injection of PEG induced intraocular activation of the complement system, which caused induction and progression of CNV in C57BL/6 mice [54].

### 2.1. Non-specific protein binding and complement responses

**Fig. 1.**
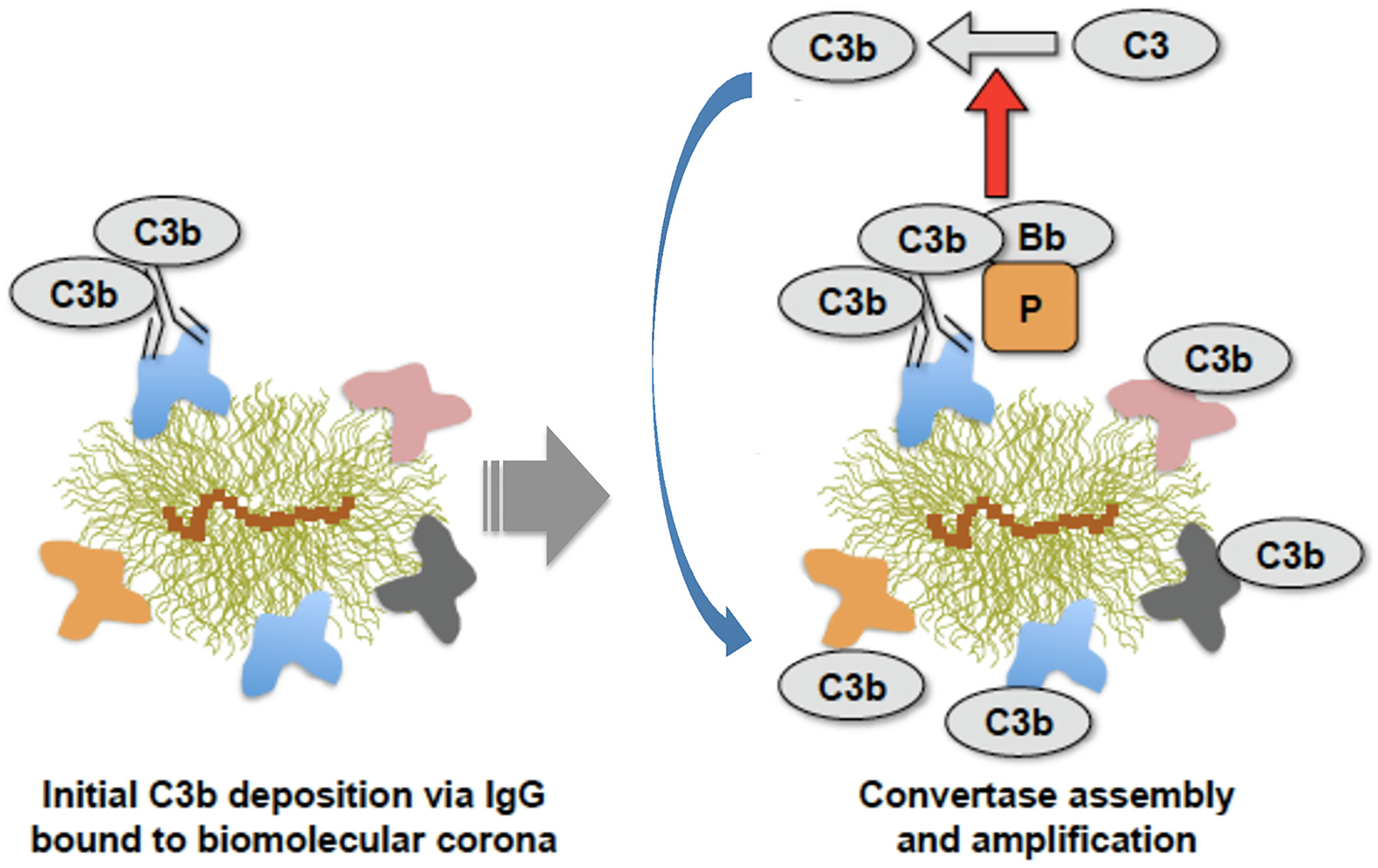
Proposed scheme for the role of biomolecule corona and immunoglobulins in activation of the alternative pathway. IgG molecules binds to the biomolecule corona, but only a few of these IgG molecules might be susceptible to attack by sponta-neously formed (nascent) C3b molecules. This results in the formation of C3bBb-properdin (i.e., the alternative pathway convertase) that initiates the cleavage of the nearby C3 molecules. We term these complement-activating nanoparticles, “catalytic nanoparticles”. Accordingly, newly generated C3b molecules bind covalently to the reactive groups (e.g., primary amines and hydroxyl moieties) of surface adsorbed proteins (amplification). Catalytic nanoparticles could inadvertently promote C3 opsonisation on the nearby nanoparticles. In addition to IgG, other immunoglobulin types may potentially trigger the process. Catalytic nanoparticles could also derive following deposition of IgG–C3b complexes that are already formed in the fluid phase and with the ability to assemble alternative pathway convertases. After Vu et al. with permission [71].

Again, early investigations with liposomes and polymeric nanospheres conclusively demonstrated that following contact with serum or plasma many proteins deposit on particle surfaces [55–61]. Recent proteomic studies have confirmed and advanced this phenomenon, also showing deposition of various complement proteins on nanoparticles [62–65]. Proteomic studies often interpreted surface deposition of complement proteins as evidence of complement activation. Such interpretations may not necessarily hold, since complement proteins (or their cleavage products) might non-specifically deposit on nanoparticle surfaces (reviewed in [66]). More concern arises with proteomic studies that have used uncharacterised serum/plasma (i.e., in terms of complement protein composition and functionality of all three complement pathways) or plasma obtained from blood collected in the presence of anticoagulants that chelate calcium and magnesium (e.g., citrate and EDTA) [66,67]. These chelators, usually at concentrations used in blood collection, suppress, modulate or inhibit complement activation [66,67]. Furthermore, the chelation process could artificially manipulate the pattern and the type of protein adsorption to nanoparticles. This is particularly important when considering a possible role for surface deposited proteins in modulating complement responses through conformational changes. Thus, meticulous handling and characterisation of sera and plasma is needed for studying complement responses [66,67]. In our experiment, we used dextran-coated superparamagnetic iron oxide nanoworms (SPION) incubated in human sera and human lepirudin-plasma [68,69]. Lepirudin is an anticoagulant that does not manipulate complement activity as its activity is through direct binding and irreversible inhibition of thrombin [70]. Through this approach, we found covalent attachment of C3/C3 opsonising fragments to surface adsorbed/intercalated blood proteins and not to the dextran shell, thereby confirming the dominant role for surface-associated proteins in directing complement responses [68]. We also showed that C3 opsonisation was reversible, indicating dynamic protein adsorption–desorption processes at surfaces [68]. In a follow up study [71] we identified a critical role for natural antibodies (e.g., IgG) in C3 opsonisation. This not only applied to SPION, but also to different regulatory-approved liposome medicines [71]. However, studies with C1 inhibitor indicated that complement activation was predominantly through the alternative pathway [71]. Interestingly, the majority of C3/C3 fragments bound to other surface-associated proteins rather than to the immunoglobulins [71]. From the analysis of C3 to IgG ratios, it was concluded that only a small fraction of nanoparticles could catalytically drive complement activation [71]. Considering the suggestion that some antibodies could trigger complement activation via the alternative pathway [72], we proposed a scheme to explain these results as depicted in Fig. 1. In order to overcome complement activation/opsonisation, improved surface engineering strategies are needed either to inhibit (or dramatically suppress) non-specific protein deposition, or to render surface bound antibodies functionally inactive. Towards these goals, nanoparticle surface modification through polymer pairing [73] or functionalisation with peptides that attract complement regulators (e.g., factor H) [74] or direct functionalisation with complement regulators (e, g., factors H and I) have proved effective [75].

The proposed tendency of some natural antibodies to trigger activation of the alternative pathway [72] might also be relevant to complement activation by PEGylated nanoparticles through the binding of anti-PEG antibodies [76]. Since, the majority of natural anti-PEG antibodies exhibit low affinity for PEG [77], it is unlikely that these antibodies could make multivalent engagement through their Fab regions (as in IgM) or form hexameric clusters (as in IgG) on PEGylated nanoparticles that attain necessary conformations to accommodate C1 complex and trigger activation of the classical pathway [44,45]. This suggestion is consistent with an observation where in the presence of anti-PEG antibodies, liposome lysis, through the assembly and insertion of the complement membrane-attack complex, was predominantly dependent on complement activation through the alternative pathway [78].

### 2.2. Complement responses to early generation dendrimers

Complement pattern-recognition molecules such as C1q and MBL can sense diverse patterns and trigger complement activation through the classical and lectin pathways, respectively [36,79,80]. Previous efforts indicated that structural and geometrical restrictions of C1q and MBL allow these molecules to sense patterns with dimensions in the range of 2–20 nm [79,80]. These suggestions led us to examine complement activation by dendrimer generations of <6 nm in size (i.e., dendrimers up to generation 5), since the angstrom-scale spacing arrangement (ASSA) of surface functional motifs (such as pyrrolidone, carboxylic acid and amine) in dendrimers might escape sensing by C1q and MBL [81]. Thus, we showed carboxylic acid- and pyrrolidone-terminated poly(amido amine) dendrimers escape surveillance by the complement pattern-recognition molecules C1q and MBL [81]. As such, these dendrimers did not trigger complement activation at all. On the other hand, while amine-terminated dendrimers could also escape sensing by the complement pattern-recognition molecules, complement became activated [81]. Here, complement activation was dependent on dendrimer hitchhiking on a subset of natural IgM glycoforms and occurred through the MBL arm of the lectin pathway [81]. This finding was consistent with earlier studies showing that IgM can trigger complement activation through MBL [82,83]. Thus, the ability of pyrroli-done- and carboxylic acid-terminated dendrimers to escape complement activation opens new opportunities for design and engineering of complement-safe nanomedicines and medical platforms [81]. Based on these findings, in the large-scale CosmoPHOS-nano project, funded by the European Union’s Seventh Framework Programme, we developed complement-safe dendrimeric nanomedicines to positively impact atherosclerosis treatment. More specifically, these dendrimer-enabled systems were designed to stabilise and passivate the vulnerable atherosclerotic plaques by preventing their rupture and acute thrombosis. We shall report the results of this study in rabbit models of atherosclerosis elsewhere. However, the use of complement-safe dendrimeric nanomedicines was advantageous, since intraplaque complement activation contributes to and promotes atherosclerotic plaque destabilisation and atherogenesis [84,85]. For instance, local liberation of the complement anaphylatoxins C3a and C5a contribute to intraplaque recruitment of inflammatory cells. Furthermore, C5a can also induce C5a receptor1-dependent nucleotide-binding domain-like receptor protein 3 (NLRP3) activation and subsequently enhance IL-1β and IL-18 production by local plaque macrophages [85].

### 2.3. Species disparity in complement responses

Many studies have outlined species and interspecies differences in complement activation and opsonisation [66,86–92]. Considering the important roles of complement system in the innate immunity, there is limited understanding as to whether complement activation by nanoparticles in preclinical species can resemble human response. For instance pigs have considerably lower levels of complement factors and lower activity of all complement pathways in comparison to humans [91,93]. This raises significant concern in using the porcine model to investigate, for instance, the involvement of complement in hypersensitivity reactions. Complement responses to nanoparticles are also different between mice and humans [92,94]. As an example, poly(2-methyl-2-oxazoline) coating of nanoparticles overcomes C3 opsonisation and confers long circulation properties to nanoparticles in mouse [94]. However, in human sera, C3 opsonisation of poly(2-methyl-2-oxazoline)-coated nanoparticles is highly efficient and dramatically promotes nanoparticle recognition and uptake by leucocytes and monocyte-derived macrophages [94].

**Fig. 2.**
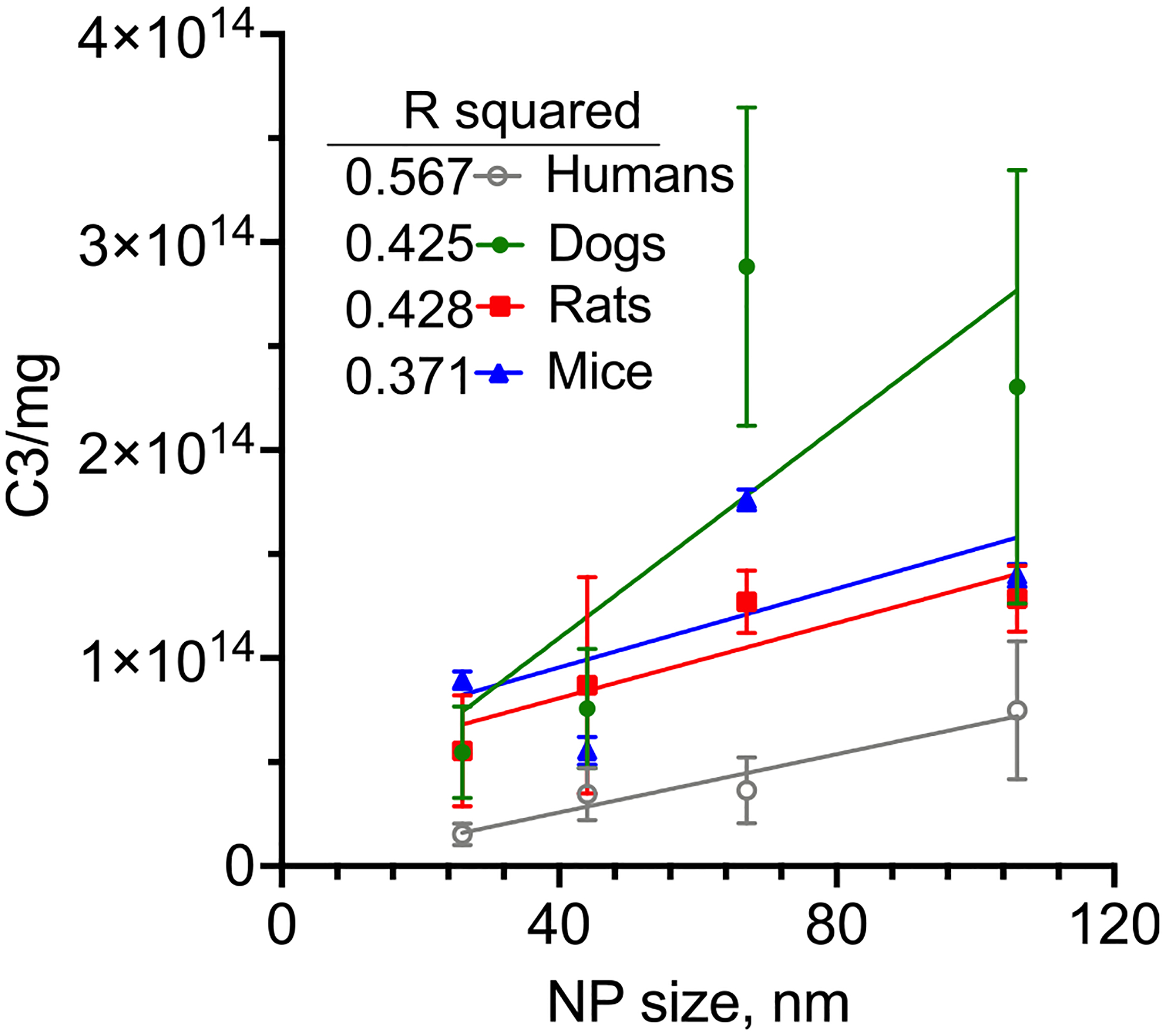
Variation among species in C3 deposition on dextran-coated superparamagnetic iron. The number of C3 molecules/mg nanoparticle increases with increasing the nanoparticle diameter in all species. After Li et al. with permission [92].

In our studies, we also compared complement responses to a panel of iron oxide nanocrystals of different sizes and dextran coatings in sera of different species. For instance, the level of C3 opsonisation (number of C3 molecules per mg Fe) in human sera is lower than that in mouse, rat and dog sera (Fig. 2) [92]. However, this study demonstrated that nanocrystals with a higher C3 deposition in dog and rat sera also displayed a higher C3 deposition in human sera, and vice versa, despite differences in the complement pathway activation and opsonisation efficiency [92]. Thus, inter- and intra-species differences and discrepancies in complement responses should be seriously considered for translational purposes and when designing haemo- and immune-compatible nanomedicines for human use.

## 3. Complement responses to archaeal viruses and a “pseudoviral” nanoplatform

**Fig. 3.**
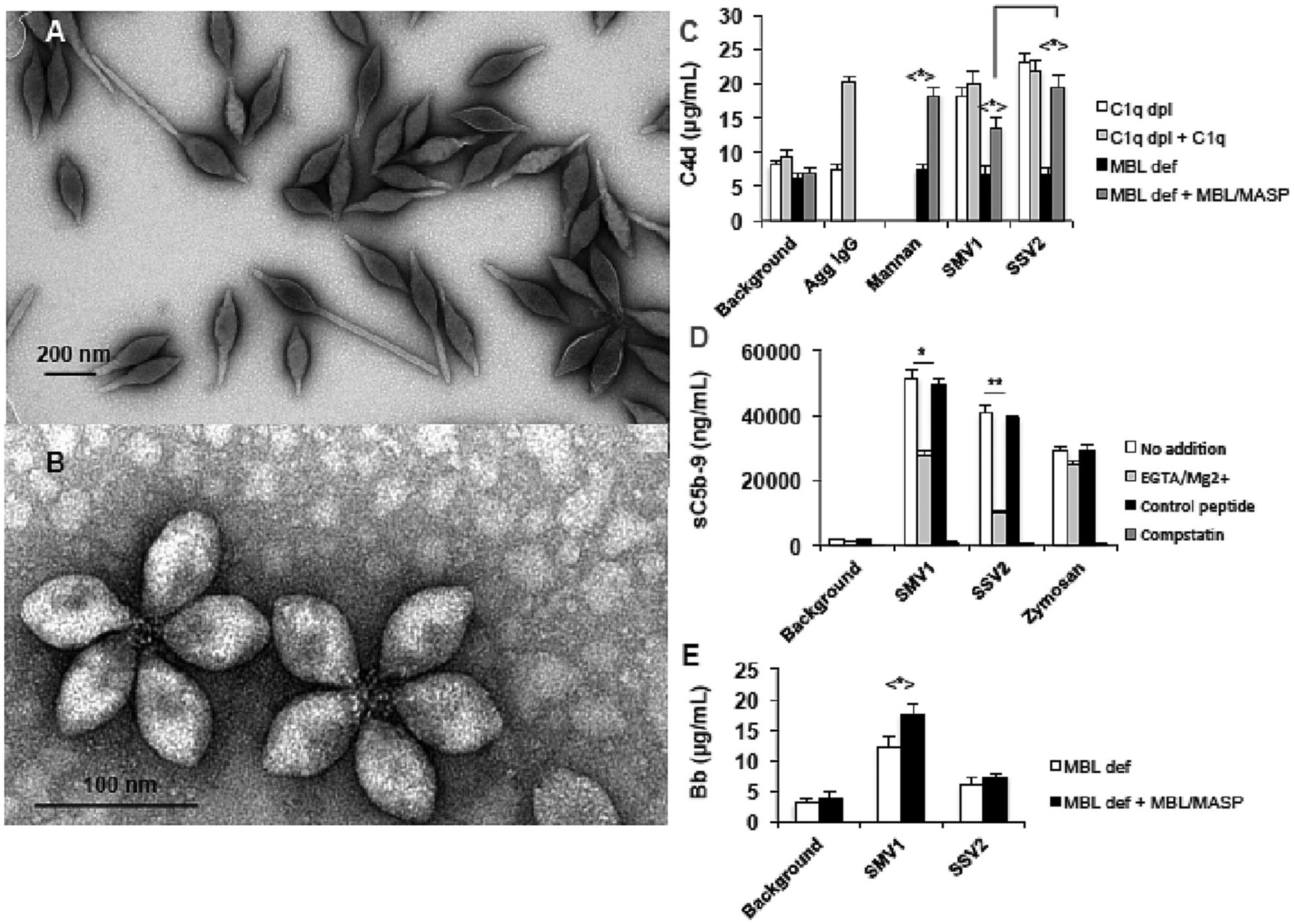
Complement activation by two archaeal viruses (SMV1 and SSV2) in human sera. A & B: transmission electron micrographs of SMV1 and SSV2, respectively. C: virus-mediated generation of C4d (a split-product of the forth complement protein) in a serum immunochemically depleted of C1q (C1q dpl) and a genetically deficient MBL serum (MBL def). D: The effect of 10 mM EGTA/2.5 mM Mg^2+^ (to inhibit calcium-sensitive complement pathways) and C3 inhibitor compstatin and its negative control peptide (40 μM) on viral-mediated activation of the terminal pathway of the complement system. E: The effect of viruses on the alternative pathway turnover (through measurement of Bb, a split-product of factor B) in MBL deficient serum and after the addition of purified MBL/MASP (MBL-associated serine proteases) equivalent to 3.0 μg MBL/mL serum. In C–E, incubations contained 588 × 10^8^ viruses. *p < 0.05; **p < 0.01. After Wu et al. with permission [97].

Extremophilic archaeal viruses are notable species that appear with distinct morphologies (e.g., droplet and bottle shapes) and remain stable under very aggressive conditions comprising very low acidic pH (<3) and high temperatures (>80 °C) [95]. There are no reports of integration of archaeal viruses into human or any other eukaryotic genomes [96]. Thus, by considering their aforementioned unique characteristics and stability, these viruses could serve as potential candidates for the development of biosensors and medical nanoplatforms. However, from an evolutionary point of view, archaeal viruses may have not developed strategies to escape sensing by the mammalian complement system. Accordingly, prior to engineering of archaeal viruses for in vivo applications, it is imperative to study their properties in relation to complement and phagocytic cell responses. Working with two such viruses we found extensive complement activation in human sera [97]. The data in Fig. 3 reports human complement responses through lectin and alternative pathways to two archaeal viruses (*Sulfolobus* monocaudavirus 1, SMV1, and *Sulfolobus* spindle-shaped virus 2, SSV2). However, the complement regulator factor H had no affinity for the viral surface and its deposition was purely C3-dependent [97]. This suggested that unlike virulent viruses [98], these species do not acquire factor H for protection. We also showed that C3 deficiency had no effect on the viral clearance by the liver and the spleen, but the splenic deposition was significantly higher in C3 knockout mice [97].

**Fig. 4.**
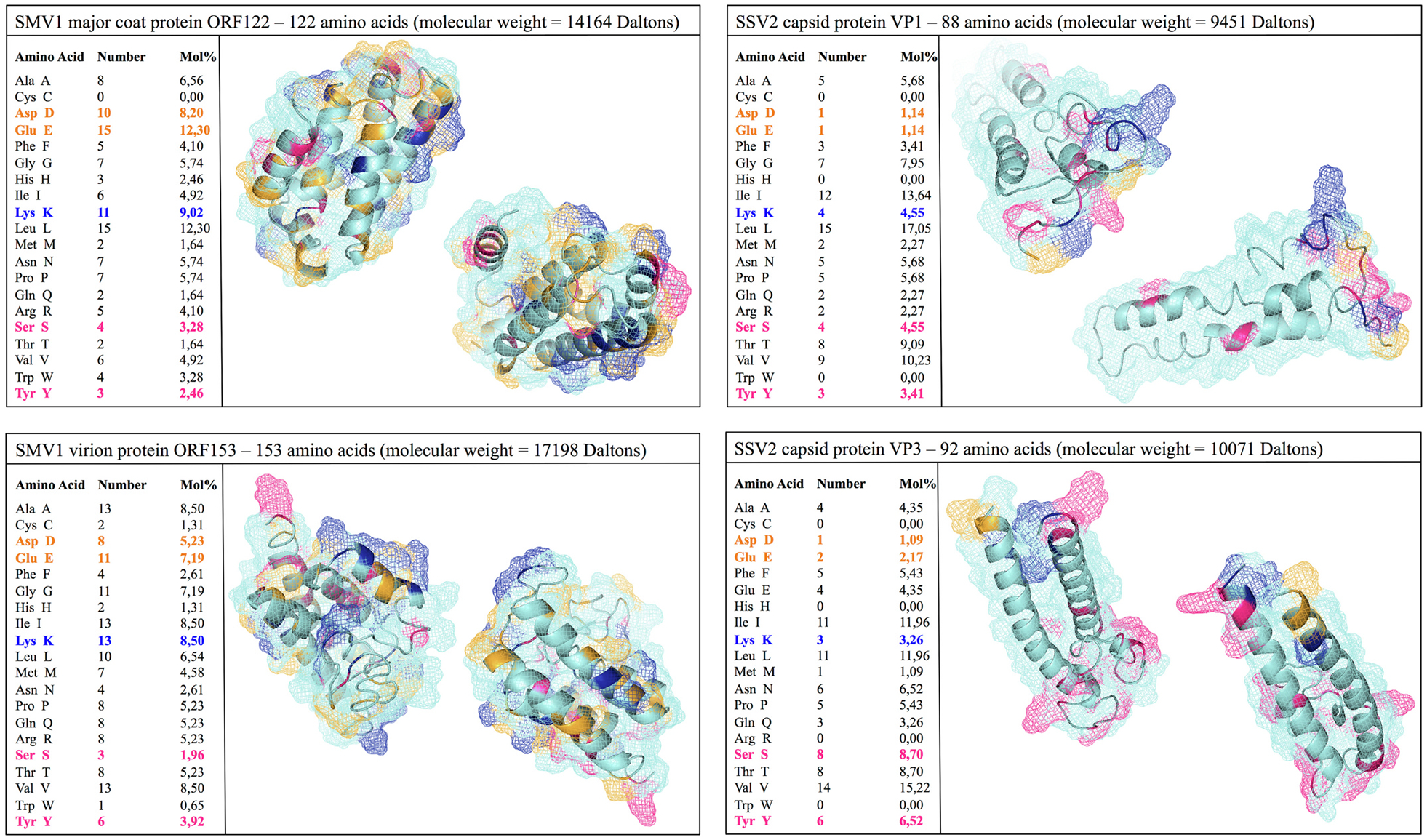
Prediction of 3-dimensional (3D) structure of the capsid proteins of SMV1 and SSV2 from their respective amino acid sequences by iterative threading assembly refinement (I-TASSER). SMV1 has two identified coat proteins (ORF 122 and ORF 153), whereas the identified coat proteins of SSV2 includes VP1 (ORF 88b) and VP3 (ORF 92). The I-TASSER server is an integrated platform for automated protein structure and function prediction based on the sequence-to-structure-to-function paradigm. Starting from an amino acid sequence, I-TASSER first generates three-dimensional atomic models from multiple threading alignments and iterative structural assembly simulations. The function of the protein is then inferred by structurally matching the 3D models with other known proteins. After Wu et al. with permission [97].

As to their potential applications in medicine, strong complement activation by archaeal viruses may provide opportunities for studying disease processes, for instance, by inducing compartmental complement activation and assessing the role of complement in disease processes and progression. Furthermore, the capsid proteins of SMVI and SSV2 express accessible lysine, aspartic acid and glutamate (Fig. 4), which could chemo-selectively be modified with ligands and polymers to engineer multifunctional biosensors and to further modulate immune responses.

**Fig. 5.**
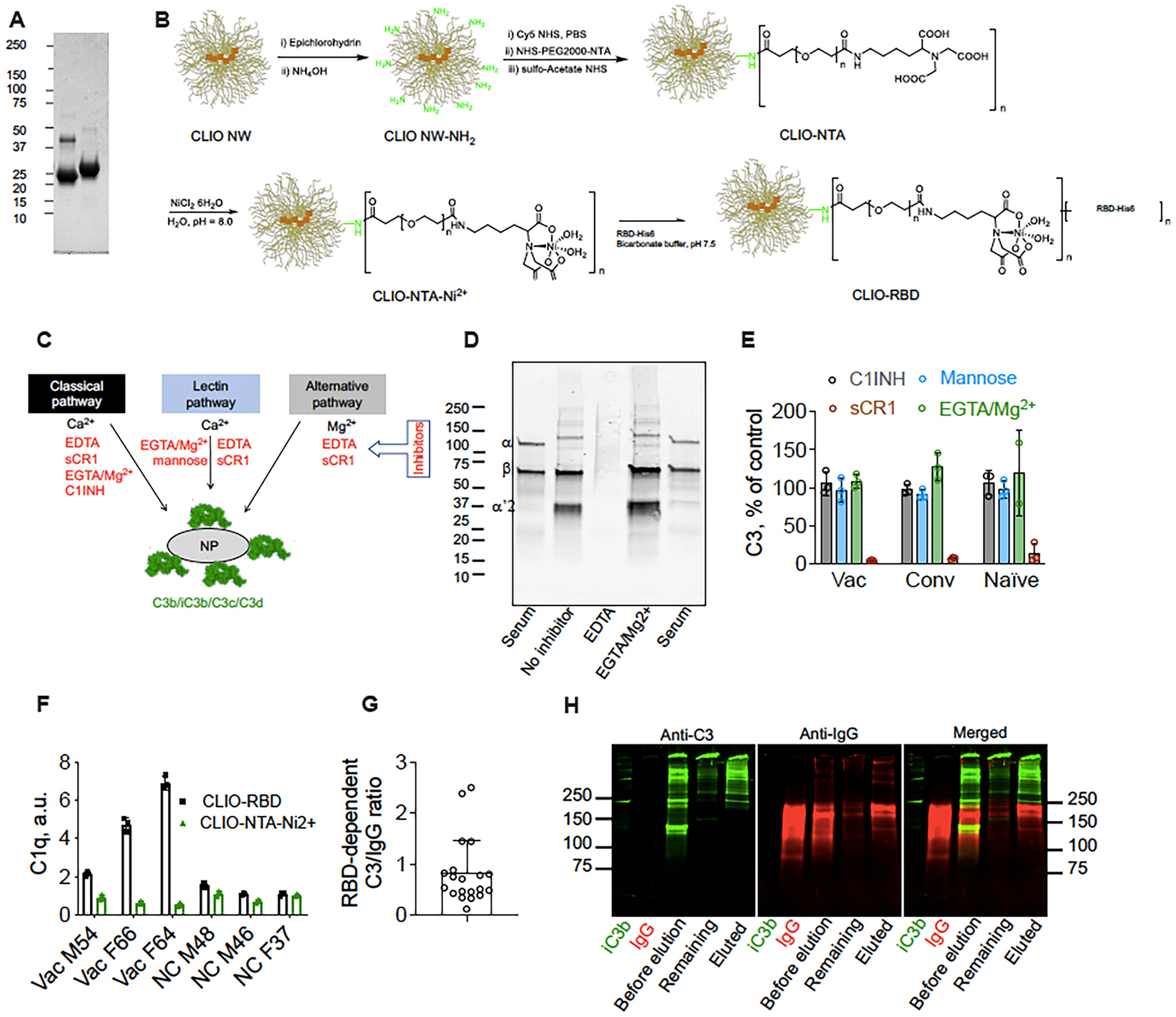
Pseudovirus-like nanoparticles for assessing complement opsonisation towards SARS-CoV-2 receptor-binding domain (RBD) in sera of vaccinated, convalescent and naïve donors. A: Purified His-tagged RBD (from left to right: nonreduced and reduced forms, respectively). B: Synthetic steps starting from cross-linked dextran iron oxide nanoworms (CLIO NWs) to CLIO-RBD. C: Schematic representation of the three complement pathways. These three complement pathways converge into C3 cleavage and nanoparticle opsonisation by C3 fragments (C3b/iC3b/C3c/C3d). Inhibitors for each pathway are shown in red. D: Western blot analysis of nanoparticle-deposited C3 in vaccinated serum. Lane 1 = serum 1:200 dilution shows native C3; Lane 2 = CLIO-RBD after incubation in serum; Lane 3 = after incubation in serum/EDTA; Lane 4 = SPIO NW after incubation in serum/EGTA/Mg^2+^. E: Complement inhibition results (% of serum control) in donors with the highest RBD-dependent C3 deposition (means of 3 donors per group, 3 technical replicates per donor) showing that the classical and lectin pathways are not involved in C3 opsonisation. C1 inhibitor (C1INH) = 100 μM; soluble complement receptor 1 (sCR1) = 1 μM; mannose = 250 μM. F: Dot-blot analysis of C1q binding, showing increased C1q binding to CLIO-RBD in vaccinated (Vac) sera as compared with naïve control (NC). However, C1q binding was extremely low and did not lead to activation of the classical pathway. G: Molar ratio of RBD-dependent C3 over RBD-dependent IgG deposition for vaccinated and convalescent donors showing a relatively inefficient enhancement of complement opsonisation. H: Analysis of association between C3 and IgG on nanoparticles in vaccinated serum (Vac M54). Proteins were eluted with 5% sodium dodecyl sulphate (SDS) and the eluted fraction and the nanoparticle-bound fraction were run in non-reducing SDS-PAGE and analysed by anti-IgG/anti-C3 Western blot. C3 in the eluted fraction is mostly not associated with IgG but appears to be bound to other proteins (higher molecular weight bands, >250 kDa). After Gaikward et al. with permission [99].

In a different programme, we engineered pseudovirus-like nanoparticles to determine some aspects of the innate immune responses to SARS-CoV-2 [99]. This initiative was to overcome challenges associated with the purification and handling of pathogenic virions. Thus, we engineered pseudovirus-like nanoparticles bearing ~70 copies of functional recombinant receptor-binding domain (RBD) of SARS-CoV-2 and monitored complement responses in sera of vaccinated, convalescent, and naïve individuals (Fig. 5) [99]. Complement fixation was highest in vaccinated and convalescent donors with the highest titres of anti-RBD IgG antibodies. However, C3 opsonisation was inefficient, and on average, each bound antibody promoted the binding of less than one C3 molecule [99]. Strikingly, the C3 molecules found bound to protein deposits and not to the IgG on the nanoparticle surface [99]. We also identified some naïve donors with relatively low affinity natural antibodies, which promoted RBD-dependent C3 deposition [99]. Nevertheless, complement fixation was antibody driven and exclusively through the alternative pathway in all cases. Complement fixation was also a prerequisite for nanoparticle uptake by granulocytes and monocytes in the blood of vaccinated donors with high anti-RBD titres [99]. These observations highlighted the heterogeneity of complement opsonisation and leucocyte uptake, which could affect the ability to clear viral infections and modulate the severity of the COVID-19. Thus, it is plausible that “high complement activators” could have a different susceptibility to SARS-CoV-2 infections or have different outcomes. One could extend these approaches to probe the affinity of anti-RBD responses to some of the COVID-19 variants and map complement opsonisation efficiency.

## 4. Complement and immune responses to brain-specific nanoparticles

**Fig. 6.**
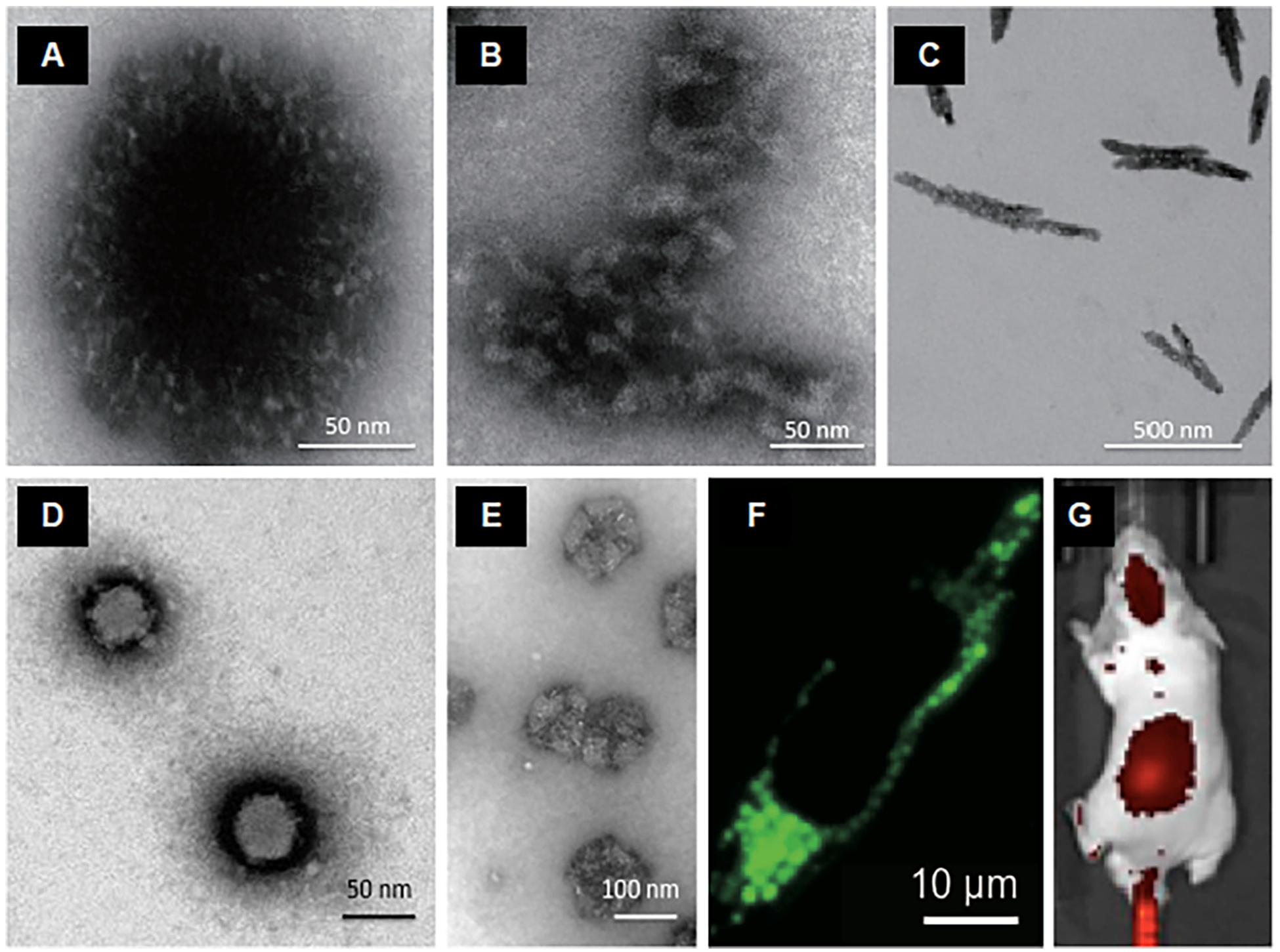
Transmission electron micrographs of NLCs and their biological performance. A & B: Native NLCs displaying unique core-shell and fiber morphologies. C, D & E: NLC-siRNA complexes. Three different morphologies are shown (rod, sphere and web-like nanostructures). F: Internalized NLCs (green fluorescent) by a human cerebral capillary endothelial cell. The uptake is through transferrin receptor and the receptor for advanced glycation end-products. G: Mouse brain localization of NLCs carrying fluorescently-labelled siRNA at 4 h post intravenous injection. Immunofluorescent sections of the brain confirmed NLC localization to the cerebral endothelial cells, neurons and microglia, but not to astrocytes. After Wu et al. with permission [102].

Development of non-viral delivery systems that can cross the blood-brain barriers is of considerable interest [100]. The brain can locally produce many complement proteins and express many other factors of the innate immunity. Thus, it is important that brain-specific nanoparticles do not trigger pro-inflammatory reactions in the brain [101]. Towards these developments, recently we introduced NanoLigand Carriers (NLCs), which are self-assemblies from a phage display conjugate peptide that targets two receptors on cerebral capillary endothelial cells (the transferrin receptor and the receptor for advanced glycation end-products) [102]. NLCs also form complexes with nucleic acids such as siRNA, mRNA and DNA (Fig. 6). We have shown the capability of these NLCs to rapidly cross the blood-brain barrier following intravenous injection and reach the neurons and microglia cells [102]. Indeed, a single intravenous injection of NLCs with functional siRNA was able to down-regulate the expression of target molecules in the brain by approximately 50% [102]. Moreover, NLCs neither induced complement activation in serum nor caused inflammatory reactions (no cytokine release and no activation of astrocytes and microglia cells in the brain) [102].

Thus, NLCs overcome previous limitations in active targeting with drug carriers decorated with phage display peptides [103]. Considering that neurological disorders are growing in incidence faster than any other disease class worldwide [104], the aforementioned noninflammatory NLCs have the potential to address shortfalls in treatment of a broad range of central nervous system diseases and disorders such as brain tumours and infections, Parkinson’s disease, Alzheimer’s disease, Huntington’s disease and amyotrophic lateral sclerosis. More specifically, NLCs may allow for minimally invasive combination drug delivery to the brain through an intravenous route of injection. This is advantageous over adeno-associated viruses (e.g., AAV9) packaged with therapeutic agents and lipid nanoparticles with cationic and ionisable lipids, which are pro-inflammatory [105,106], as well as over therapeutics administered into the cerebrospinal fluid, which are also not without risks and serious adverse reactions [104].

## 5. Infusion reactions to nanoparticles and nanopharmaceuticals

**Fig. 7.**
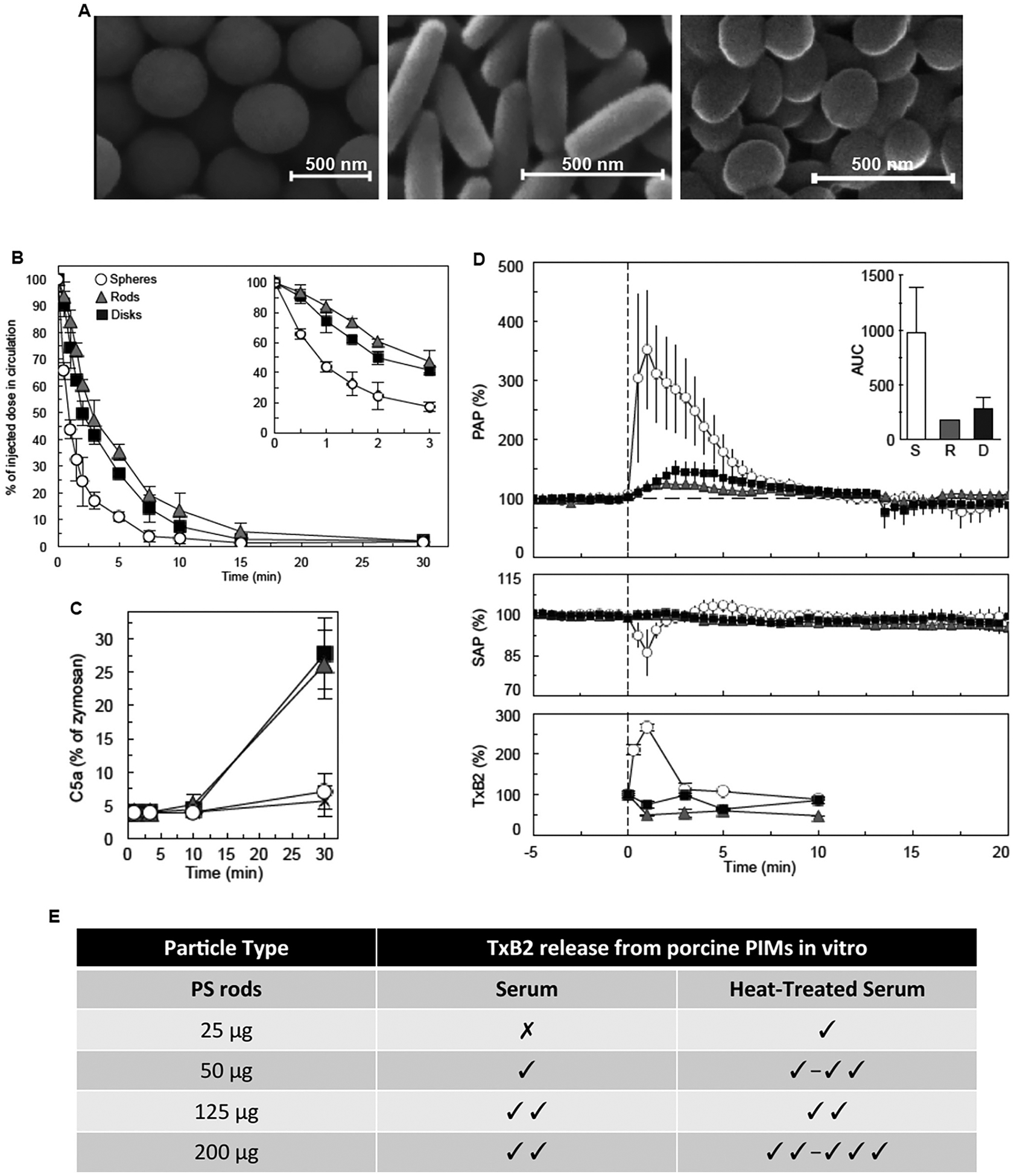
Porcine responses to intravenously injected carboxylated polystyrene nanoparticles with different shapes (spheres, rods and disks). A: Scanning electron micrographs of carboxylated polystyrene spheres (500 nm), rods (450 × 120 nm) and disks (250 × 75 nm). B: Circulation profile of rhodamine-labelled nanoparticles following intravenous injection into pigs. Nanoparticles were injected at a dose of 1.5 × 10^11^ nanoparticles per 20 kg body weight. The spheres are cleared faster than the rods and the disks. *p* < 0.05 (non-paired two-sided *t*-test comparing spheres with rods and disks) for all points between 30 s and 3 min. C: Time-dependent complement anaphylatoxin C5a generation in pig whole blood on exposure to nanoparticles relative to 0.2 mg mL^‒1^ zymosan response. Complement activation by polystyrene nanoparticles was compared on an equivalent surface area of ~14,500 nm^2^ per mL of blood. D: Time-dependent changes in pulmonary arterial pressure (PAP), systemic arterial pressure (SAP) and thromboxane B2 (TxB2) release on nanoparticle injection (~114,300 nm^2^ per 20 kg body weight) into pigs compared with background (resting phase, before 0 min). Inset = integrated area under the curve (AUC) of the changes in PAP during the first 10 min of injection. E: TxB2 release by isolated pig PIM cells following challenge with rod-shaped polystyrene nanoparticles in the presence of autologous fresh and complement inactivated serum (heat-treated at 53 °C, 90 min). Panels A–D, after Wibroe et al. with permission [111].

**Fig. 8.**
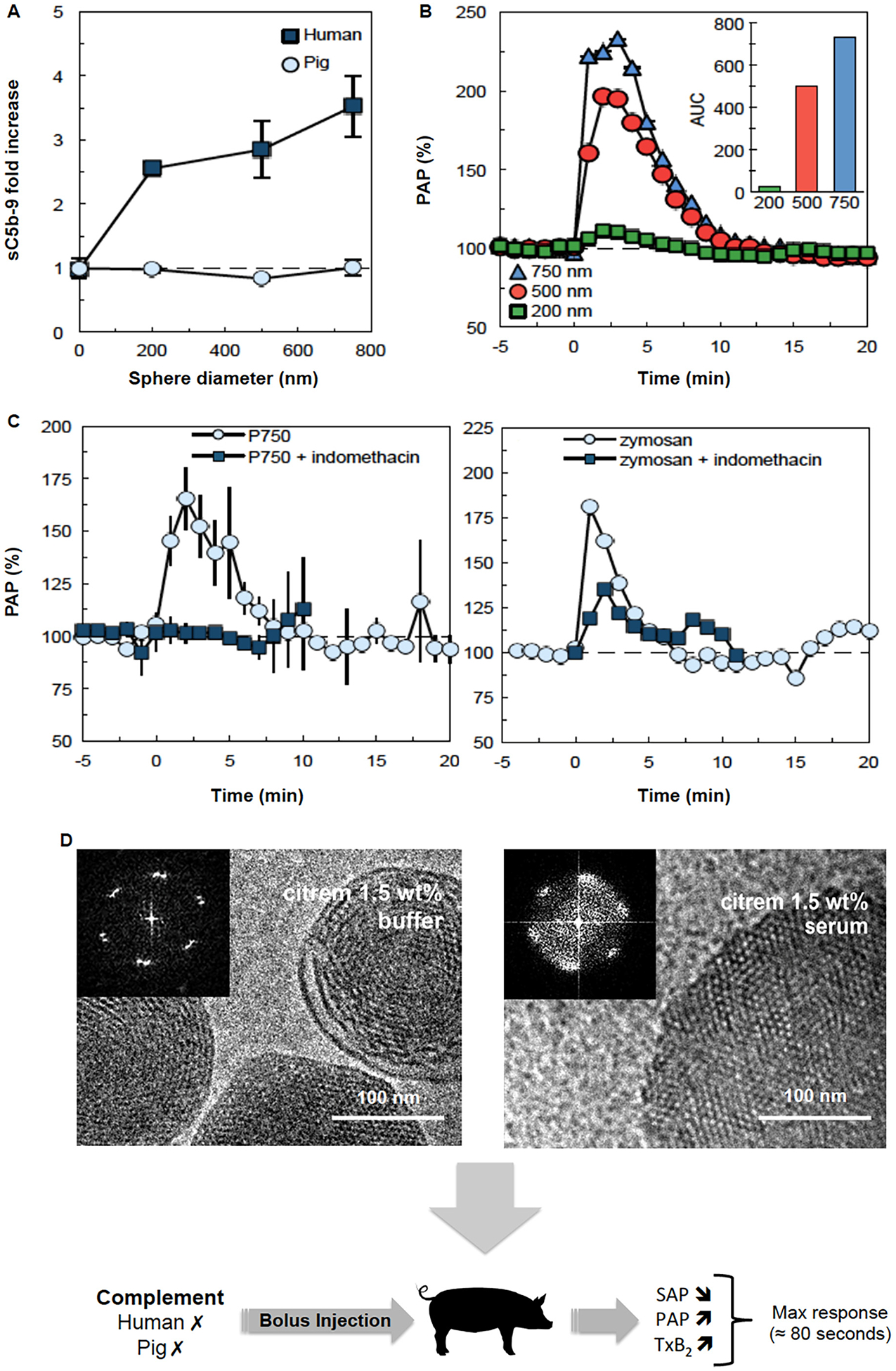
Cardiopulmonary distress to polymeric nanoparticles and lipidic citrem-stabilised hexosomes. A: Complement activation (as a measure of sC5b-9) in human and pig whole blood after 30 min exposure to sulfated polystyrene nanoparticles of different sizes. Comparison was made at an equivalent surface area of ~14,500 nm^2^ per mL of blood. Measurement of sCb5-9 was in accordance with an established enzyme immunoassay procedure responsive to human and porcine serum/plasma [111,135]. Zymosan-activated porcine plasma (with lepirudin as anticoagulant) showed 11-fold increase in sC5b-9 levels over background. B: Size-dependent nanoparticle-mediated rises in pulmonary arterial pressure (PAP) in pigs. Comparison was made at an equivalent nanoparticle surface area of ~114,300 nm^2^ per 20 kg body weight. Resting phase is before 0 min. Inset = integrated area under the curve (AUC) of the changes in PAP during the first 10 min of injection. C: Attenuation of the carboxylated polystyrene nanoparticles (750 nm in diameter) after inhibition by 1 mg kg^‒1^ indomethacin. These nanoparticles are mild activators of porcine complement and injected at a dose of 1.69 × 10^9^ spheres per kg body weight. A low dose zymosan (1 mg kg^‒1^) response also induces PAP rises but prior indomethacin injection partially reduces zymosan-mediated PAP rises. D: Pigs also undergo cardiopulmonary distress within minutes of intravenous injection of hexosomes made from glyceryl monooleate and citrem (an anionic citric acid ester of monoglycerides), which is used as a stabiliser at a concentration of 1.5 wt%. These hexosomes do not trigger complement activation in either human (sC5b-9 and C5a measurements [136]) or porcine blood collected in the presence of anticoagulant lepirudin (C5a measurement using a commercially available porcine-specific ELISA kit). The panel also shows cryogenic transmission electron micrographs of hexosomes before (prepared in phosphate buffered saline) and after exposure to serum. Corresponding fast Fourier transform analysis of nanoparticles’ interiors are shown as insets. Panel C: after Wibroe et al. with permission [111]. Electron micrographs in panel D: after Wibroe et al. with permission [136].

Another important subject is the reported infusion reactions to nanomedicines such as PEGylated liposomes (e.g., Doxil^®^), non-PEGylated liposomes (e.g., Ambisome^®^), lipid nanoparticles (e.g., Onpattro^®^), and iron-based nanoparticles (e.g., intravenous iron preparations) in human subjects, which are idiosyncratic and apparently non-IgE-dependent. This subject has been repeatedly reviewed and discussed with different views (reviewed in [22,26,35,107]). For instance, one hypothesis suggests that following nanomedicine-mediated complement activation, liberated anaphylatoxins (e.g., C3a and C5a) trigger adverse haemodynamic and cardiopulmonary reactions [34,108]. However, there is no conclusive evidence to support this hypothesis in human subjects. If complement is involved in hypersensitivity reactions to nanopharmaceuticals, then kinetics of anaphylatoxin generation and inactivation could be a rate-limiting factor in defining the response magnitude. Another rate-limiting factor could be individual variations in anaphylatoxin receptor threshold. Severe nanopharmaceutical-mediated cardiopulmonary distress [i.e., rapid rise in the pulmonary arterial pressure (PAP) and a simultaneous drop in the systemic arterial pressure (SAP)] is outwardly demonstrable in pigs and occur within a few seconds of injection [108–110]. In a porcine study, we also demonstrated cardiopulmonary distress (as alterations in PAP and SAP changes and thromboxane B2 release) immediately on injection of carboxylated and sulfated polystyrene nanoparticles, PEGylated liposomes and zymosan [111] (selected examples are presented in Figs. 7 & 8). However, complement activation in vitro (as a measure of C5a release) by nanoparticles (at a surface area of 14,500 mm^2^ per mL of blood) in the blood of the same animals indicated that the extent of C5a liberation was <5% of that generated by zymosan, within the first 10 min of incubation (Fig. 7), but in vivo the magnitude of cardiopulmonary distress was comparable between nanoparticle and zymosan administration [111]. Elsewhere, others showed that a low dose injection of a recombinant C5a (330 ngKg^‒1^) to pigs, which immediately raised plasma C5a levels by 40% over the baseline, only induced a mild reaction with reversible hypertension [108]. In contrast, a large dose of C5a (440 μgKg^‒1^) raised blood C5a by 600–700-fold and caused a short-lived transient hypertension, which immediately followed by massive hypertension, bradyarrhythmia and pulmonary hypertension [108]. The high-dose C5a responses mimicked rapid cardiopulmonary distress seen with liposomes (0.1 mgKg^‒1^ Doxil^®^ and 0.5 mgKg^‒1^ multi-lamellar vesicles, respectively) and zymosan administration [108]. In contrast to the in vivo observation with the high dose C5a injection, in vitro experiments in porcine plasma with Doxil^®^ and multi-lamellar vesicles (5 mg phospholipid/mL) raised C5a levels by approximately 4- and 6-fold above the background, respectively, after 15 min of incubation [108]. The reasons for these discrepancies are not clear, but it may be related to plasma preparation and handling or alternatively to the mild porcine complement responses to lipid nanoparticles (at least in vitro). Nevertheless, considering that apparently several hundredfold rise of plasma C5a in pigs is necessary to induce severe cardiopulmonary distress, the question is whether Doxil^®^ (or other nanopharmaceuticals) could liberate such high levels of anaphylatoxins, particularly within a few seconds of injection. Furthermore, this study [108] neither investigated the effect of intermediary doses of C5a on cardiopulmonary distress nor inactivation/clearance rate of C5a in pigs. Thus, it is difficult to conclude to what extent complement and its activation kinetics is important in directing hypersensitivity reactions to nanopharmaceuticals in the porcine model. A role for C3a in severe reactions in pigs is also possible, but this was not investigated either. Towards resolving the role of complement anaphylatoxins in infusion reactions, we have not been able to inhibit complement in pigs in vivo, since availability of porcine-specific complement inhibitors and C3 knockout pigs are scarce [112,113]. Notwithstanding, considering differences between porcine and human complement functionality [91], we should further await studies with complement therapeutics (e.g., complement inhibitors of different steps of the complement cascade either approved by regulatory bodies or in clinical development) [114–116] to evaluate and dissect the role of complement cascade in nanomedicine-mediated infusion reactions in humans.

The second hypothesis considers a role of macrophages (and other immune cells) as effector cells in hypersensitivity to nanoparticles, at least in the porcine model [26]. Unlike a normal human lung, pigs have pulmonary intravascular macrophages [117,118]. Early studies have shown that pigs, and other animals that have resident macrophages in their lung vasculature (e.g., sheep), respond to intravenously injected particles (and endotoxin), where rapid particle (or endotoxin) clearance by PIMs correlate with the peak periods of the cardiopulmonary distress, including vasoconstriction, bronchoconstriction and pulmonary hypertension [119,120]. Indeed, PIMs are a major source of the arachidonate metabolites, and administration of the cyclooxygenase inhibitor indomethacin abrogates pulmonary haemodynamic responses seen on particle injection [117]. Newborn lambs do not have PIMs, but PIMs appear within 2 weeks of birth [120]. Furthermore, newborn lambs do not undergo pulmonary hypertension on nanoparticle injection, but a transient pulmonary hypertension to nanoparticle administration occurs when PIMs populate the lungs [120]. These observations, strongly suggest an important role for PIMs as effector cells in directing cardiopulmonary distress, particularly on robust nanoparticle clearance from the systemic circulation. Collectively, these observations have generated controversy regarding pig as a valid model for studying human infusion-reactions to nanopharmaceuticals [22,26,43,109,110,121–125].

Our own studies in pigs also support an effector role for PIMs in infusion reactions to nanoparticles [111]. For instance, Figs. 7 & 8 summarise the results of cardiopulmonary responses in pigs following intravenous injection of a wide range of nanoparticles. The first notable observation is not all nanoparticle types trigger cardiopulmonary distress immediately on injection. For instance, deviations from a spherical shape by the same material (e.g., discs and rods) overcome adverse reactions [111]. This observation is in line with the initial significantly slower clearance rate (within the two minutes) of rods and discs from the blood when compared with spheres. With spherical shape, size plays an important role in modulating adverse responses for the same material. Fig. 8 shows that pigs dramatically respond to an intravenous injection of larger sulfated polystyrene nanoparticles (≥500 nm) than their smaller counterparts (200 nm) when injected at an equivalent surface area, although none of these nanoparticles trigger complement activation in the porcine blood. This equivalent nanoparticle surface area also translates to an approximate dosing of 16 and 43 times more nanoparticles with a size of 200 nm as compared to those with 500 nm and 750 nm sizes, respectively. Since particle size (or volume) is an important determinant in macrophage uptake, these differences, most likely, indicate a role for uptake kinetics, receptor type and signalling processes in directing PIM responses. For instance, it is plausible that marginally slower clearance kinetics could prompt desensitisation circuits [26], and we are investigating these possibilities. In line with these suggestions, attachment of larger spherical polystyrene particles to erythrocytes prior to intravenous injection also proved effective in dampening adverse reactions in the porcine model [111]. This approach also delays particle presentation to the macrophages and slows their clearance from the blood [126]. Considering the role of PIMs in infusion reactions, an intriguing question is why Doxil^®^, with its extended blood longevity (at least in rodents and humans), can trigger adverse responses in pigs [108]. To the best of our knowledge, at present, there are no reports on Doxil^®^ pharmacokinetics and biodistribution in pigs, but early studies in rodents (and humans) have demonstrated rapid clearance of otherwise long circulating particles by macrophage-rich organs, depending on pathophysiological conditions [127–129]. Furthermore, in a previous study [130] we demonstrated vesicular population curvature differences in Doxil^®^ batches and its follow-on products. Curvature differences could modulate projected PEG conformation and shielding efficacy, thereby affecting vesicular clearance by phagocytic cells (reviewed in [49]). Thus, rapid ingestion of poorly shielded vesicles by PIMs through a signalling receptor might explain adverse reactions to Doxil^®^.

The second important observation from our studies arises from nanoparticles that do not trigger porcine complement (at least in vitro). These nanoparticles also initiated cardiopulmonary distress immediately on intravenous injection. For example, we showed complement insensitivity of a batch of commercially available sulfated polystyrene nanospheres of different sizes as well as a class of hexosomes in the porcine blood (positive controls included Doxil^®^ and zymosan), whereas on intravenous injection these nanoparticles induced cardiopulmonary distress (Fig. 8). In line with in vivo studies, experiments with isolated PIMs further show that nanoparticles promote mediator release (e.g., thromboxane B2) even in the absence of functional complement (heat-denatured serum, 53 °C/90 min). However, the extent of thromboxane B2 release from PIMs is dependent on the nanoparticle dose (Fig. 7).

More support for the role of PIMs in adverse reactions to nanoparticles and other agents (e.g., zymosan and endotoxins) comes from in vivo experiments following PIM destruction (e.g., through administration of clodronate-loaded liposomes) or on indomethacin administration [111,131]. Indeed, PIM destruction prevents or dampens cardiopulmonary distress to nanoparticles, zymosan and endotoxin [111,131]. Nevertheless, considering the aforementioned arguments, we emphasise that “induced” PIMs have been identified in pulmonary circulation of human subjects, but highly dependent on the pathology [132–134]. Thus, it is plausible that nanomedicine-mediated infusion reactions in humans arises from the presence of either “induced” PIMs and/or a population of other responsive immune cells that reside either in lung vasculature or outside the pulmonary circulation (e.g., spleen, liver, blood).

Finally, the contact system [137,138] could also play a role in infusion reactions to nanoparticles, but this has not received much attention. For example, kallikrein-kinin system might be able to initiate adverse responses through a bradykinin-dependent mechanism [137]. High molecular weight kininogen has six domains with a range of proinflammatory and procoagulant functions [137]. Activated coagulation factor XII (Hageman factor) can activate plasma kallikrein, which, in turn, can cleave the high molecular weight kininogen causing the release of the pro-inflammatory peptide bradykinin [137,138]. Brady-kinin is known to stimulate the production of superoxide radicals and nitric oxide, and modulate the release of histamine, arachidonic acid, prostaglandin E2 and a whole range of pro-inflammatory cytokines [138]. Bradykinin, also promote neutrophil migration [138]. Theoreti-cally, these bradykinin-mediated responses fit with the cardiopulmonary distress symptoms of infusion reactions to nanopharmaceuticals [26,107]. Thus, some nanoparticles such as amorphous silica nanoparticles and certain anionic liposomes (e.g., phsophatidylserine) could potentially trigger the contact system by activating the zymogen factor XII [139]. This process either could be dependent on the pristine characteristics of nanoparticles and/or triggered by surface-adsorbed blood proteins, such as those with amyloid-like structures. Parallel with these speculations, it is also plausible that some nanoparticles could promote the release of procoagulant microvesicles from the plasma membrane of monocytes and platelets [140–142], which, in turn, might contribute to pro-inflammatory reactions. Indeed, the outer surfaces of these microvesicles are rich with phosphatidylserine, which provides a catalytic surface for the assembly of contact and coagulation factors [140–142]. Plasma kallikrein can also cleave C3 and activate the alternative pathway of the complement system [143,144], but whether this occurs in vivo is not clear.

### 5.1. Hypersensitivity in rodents: An alternative pathway of anaphylaxis

Studies by Strait et al. [145] indicated existence of an immunoglobulin-dependent (particularly of IgG1, IgG2a and IgG2b subclasses) pathway of systemic anaphylaxis in mice through the macrophage low affinity IgG receptor (i.e., FcγRIIIA). Whether this pathway operate in humans is not clear, but human macrophages express FcγRIII [146]. Notwithstanding, early in vitro experiments have shown that phagocytosis of IgG-opsonised particles, but not iC3b-opsonised particles, lead to proinflammatory reactions (e.g., upregulation of arachidonic acid and hydrogen peroxide production) in both murine and human macrophages [147,148]. However, in vitro experiments with murine primary bone marrow-derived macrophages showed significantly more proinflammatory cytokine production after complement-mediated phagocytosis when compared with FcγR-mediated uptake [149]. This proinflammatory response was apparently dependent on calpain-mediated activation of nuclear factor-κВ [149]. Accordingly, in murine, systemic anaphylaxis may also be dependent on complement opsonisation. Future experiments with complement inhibitors could test this possibility in vivo in rodents.

Finally, mouse basophils also express FcγRIIIA and play a role in IgG-mediated anaphylaxis through the release of platelet-activating factor [150]. Human basophils exclusively express activating FcγRIIA and inhibitory FcγRIIB [151]. Activating FcγR can activate both human and mouse basophils; however, with different efficacies, but negative signals triggered by the inhibitory FcγR seem to dominate positive signals triggered by activating FcγR [151].

## 6. Future development

Nanoparticles interact with the immune system in many different ways. The foregoing has described some collective efforts from our laboratories on mapping nanoparticles’ interactions with the complement system and different phagocytic cells such as neutrophils, monocytes and PIMs. Knowing how the immune system recognises and clears administered nanoparticles helps with optimising nanomedicines design for modulating immune responses per se and for treating diseases and disorders of phagocytic cells. On the other hand, escaping immune surveillance is essential for nanoparticles (and other medical platforms) to safely exert their therapeutic potentials in the body. However, through spatiotemporal changes in biological milieu and engagement with multifaceted arms of the immune system, nanoparticles (including their PEGylated counterparts) could elicit acute and chronic inflammation and initiate adverse reactions. Such complexities have also surfaced with rare episodes of anaphylactic reactions to lipid nanoparticle vaccines (which contain PEGylated lipids) in vaccinated individuals [152]. However, while some reports show elevated levels of anti-PEG antibodies in subjects vaccinated with Pfizer-BioNTech or Moderna lipid nanoparticle-based mRNA vaccines against SARS-CoV-2 [153], new studies show no obvious association between anti-PEG antibodies and adverse reactions to these vaccines [154,155].

Not only do the material properties play a modulatory role in nanoparticle interactions with the immune system, but also an inadvertent adsorption of non-specific biomolecules onto the surfaces of the nanoparticles could dynamically influence subsequent responses by the immune system. Future developments should carefully scrutinise and further study materials characteristics and map their immunological impact through systems immunology in relation to the developmental stages of diseases in validated animal models and humanised explant models that address intrinsic characteristics of different tissues. Indeed, some of the abovementioned suggestions are being investigated in the ongoing DIRNANO project, which is supported by the European Union’s Horizon 2020 programme under Marie Skłodowska-Curie Actions. Our work in progress further considers and computes inter-individual immune responses to nanomedicines and correlates these to innate immunity genes and their polymorphisms. These approaches could pave the way for patient stratification in nanomedicine-based therapies.

## Data Availability

No data was used for the research described in the article.
